# Visualization of the distortion induced by nonlinear noise reduction in computed tomography

**DOI:** 10.1117/1.JMI.10.3.033504

**Published:** 2023-06-15

**Authors:** Joel Larsson, Magnus Båth, Anne Thilander-Klang

**Affiliations:** a University of Gothenburg, Sahlgrenska Academy, Institute of Clinical Sciences, Department of Medical Radiation Sciences, Gothenburg, Sweden; b NU Hospital Group, Section of Diagnostic Imaging and Functional Medicine, Trollhättan, Sweden; c Sahlgrenska University Hospital, Department of Medical Physics and Biomedical Engineering, Gothenburg, Sweden

**Keywords:** computed tomography, image quality, noise reduction algorithms, nonlinear reconstruction

## Abstract

**Purpose:**

We developed a method to visualize the image distortion induced by nonlinear noise reduction algorithms in computed tomography (CT) systems.

**Approach:**

Nonlinear distortion was defined as the induced residual when testing a reconstruction algorithm by the criteria for a linear system. Two types of images were developed: a nonlinear distortion of an object (${\mathrm{NLD}}_{\text{object}}$) image and a nonlinear distortion of noise (${\mathrm{NLD}}_{\text{noise}}$) image to visualize the nonlinear distortion induced by an algorithm. Calculation of the images requires access to the sinogram data, which is seldomly fully provided. Hence, an approximation of the ${\mathrm{NLD}}_{\text{object}}$ image was estimated. Using simulated CT acquisitions, four noise levels were added onto forward projected sinograms of a typical CT image; these were noise reduced using a median filter with the simultaneous iterative reconstruction technique or a total variation filter with the conjugate gradient least-squares algorithm. The linear reconstruction technique filtered back-projection was also analyzed for comparison.

**Results:**

Structures in the ${\mathrm{NLD}}_{\text{object}}$ image indicated contrast and resolution reduction of the nonlinear denoising. Although the approximated ${\mathrm{NLD}}_{\text{object}}$ image represented the original ${\mathrm{NLD}}_{\text{object}}$ image well, it had a higher random uncertainty. The ${\mathrm{NLD}}_{\text{noise}}$ image for the median filter indicated both stochastic variations and structures reminding of the object while for the total variation filter only stochastic variations were indicated.

**Conclusions:**

The developed images visualize nonlinear distortions of denoising algorithms. The object may be distorted by the noise and vice versa. Analyzing the distortion correlated to the object is more critical than analyzing a distortion of stochastic variations. The absence of nonlinear distortion may measure the robustness of the denoising algorithm.

## 1 Introduction

A major advantage of computed tomography (CT) compared with conventional planar radiography is the possibility of delineating anatomical structures in three dimensions, allowing more information to be gathered from a CT examination. Historically, CT examinations have required much higher absorbed radiation doses to the patient to keep the quantum noise in the CT images reasonably low. However, recent advancements in CT technology have led to the possibility of acquiring CT images at sub-mSv radiation doses^1^^,^^2^ and even at the same dose as in conventional radiographic imaging.^3^ Improvements in noise reduction algorithms in the reconstruction of CT images have been important in this development. Recently developed algorithms are mainly based on iterative approaches and deep learning to reduce the noise in CT images.^4^^,^^5^

A clinical CT system will always exhibit nonlinear distortion due to its physical limitations as the detector elements cannot be infinitely small, and data cannot be collected continuously around the patient. In addition to leading to aliasing, these limitations, in combination with the presence of scattered radiation and a diverging beam, may create a partial-volume effect and streak artifacts.^6^ As filtered back-projection (FBP) has been the gold standard of reconstruction techniques, convolution kernels have been the only reconstruction option to balance the distortion suppression between for example, reducing streak artifacts and increasing the blurring of the image and vice versa. Iterative reconstruction is often less sensitive to abrupt divergences between adjacent projections than FBP, and the nonlinear distortion due to geometrical inconsistencies may thus be reduced.^7^ However, other distortion effects may arise from the often nonlinear behavior of the algorithms included in iterative reconstruction. For example, overregularization in a nonlinear noise reduction algorithm may lead to an unfamiliar smoothing often described as plastic.^8^ Also the regularization factors in the algorithm may adapt to the composition of the patient using prior object information modeling.^9^ Consequently, the image quality cannot be generalized as it will depend on the contrast of the imaged objects and the noise level.

The image quality of images reconstructed using nonlinear noise reduction algorithms has been evaluated by altered metrics from the theory of linear systems. For example, the concept of the modulation transfer function has been adapted to apply for specific tasks by the task-specific transfer function (TTF) as the spatial resolution may vary depending on the noise and the contrast of the imaged object.^10^^,^^11^ The TTF has further been applied in the concept of model observers to calculate a detectability index ($d^{\prime }$) for specific detection tasks.^12^ Recently, the method of TTF has been applied to patient images.^13^^,^^14^ Other approaches, such as quantifying the overregularization, have also been used to describe the performance of a nonlinear noise reduction algorithm.^8^ The performance of nonlinear noise reduction algorithms may also be characterized by decoupling the distortion from the system resolution as the distortion may masquerade as a degradation in spatial resolution.^15^^,^^16^ However, a diagnosis will often depend on the radiologist’s interpretation of the detected pathology and a nonlinear noise reduction algorithm may distort the image such that the new image impression alters this interpretation.^9^ Hence, an overview of the distortion of a reconstructed image would be useful to improve our understanding of the effects of the nonlinear noise reduction algorithm. A comparison with an FBP image is often used to demonstrate the robustness of nonlinear noise reduction algorithms.^17^ Although such a comparison may be interesting, it does not analyze the nonlinear effect of the algorithm but instead the difference between the algorithms. Furthermore, the difference will consist of both linear and nonlinear distortions induced by the algorithms, in which the absence of nonlinear distortion is a measure of robustness of the noise reduction algorithm. Thus the purpose of this study is to develop a method that can isolate and visualize the location of the nonlinear distortion caused by a nonlinear noise reduction algorithm in an arbitrary object, independently of other algorithms. Two new types of images that estimate and visualize the behavior and noise dependence of a nonlinear algorithm are proposed. The first type, denoted the ${\mathrm{NLD}}_{\text{object}}$ image, visualizes the systematic nonlinear distortion of the object at a given noise level. The second type, denoted the ${\mathrm{NLD}}_{\text{noise}}$ series (which consists of many images), visualizes the nonlinear distortion of the noise caused by the object.

## 2 Materials and Methods

The method used in this study was inspired by the theory of the distortion power spectrum (DPS) developed by Wells and Dobbins^15^ and later implemented in CT by Larsson et al.^16^ These studies investigated the waveform distortion as the transfer of power from one spatial frequency to another and used sinusoidal test objects to analyze the distortion at individual frequencies. Further, the latter study was based on forward projecting these objects to a sinogram to test nonlinear reconstruction algorithms. Although the distortion at individual frequencies may characterize a nonlinear algorithm, the distortion is dependent on the composition of the object, i.e., the distortion of an object is not equal to the sum of the distortion of the individual frequencies of the object. Hence, the present method was developed for analysis of the distortion of an arbitrary object. Further, the methods used in the mentioned studies and in the present one are basically testing the criteria for a linear system. In contrast, the present method investigates the distortion in the image domain and any changes to an object when reconstructed in low versus high noise. Only a nonlinear reconstruction will be dependent on the noise level. Hence, the present study defined the observed changes between noise levels as nonlinear distortions. The method was applied to a typical CT image and tested using simulations of the CT acquisition and reconstruction algorithm as access to projection data was limited on the existing CT systems at our disposal. Similar to Larsson et al.,^16^ the present method is based on manipulation of the sinogram. However, the ${\mathrm{NLD}}_{\text{object}}$ was approximated to not require access to the sinogram but was investigated here using simulations.

### 2.1 Description of the Method

The noise reduction algorithms used in CT image reconstruction may affect image quality nonlinearly, i.e., they may be dependent on the imaged object and the quantum noise. One of the effects may be nonlinear distortion, i.e., instead of a reduction in the signal, part of the signal of the object is transferred to other image structures. To identify the nonlinear distortion of a reconstruction algorithm, two types of images are proposed: one containing the nonlinear distortion of objects (${\mathrm{NLD}}_{\text{object}}$) and the other containing the nonlinear distortion of noise (${\mathrm{NLD}}_{\text{noise}}$). These images reveal the nonlinearity of a system by visualizing the extent to which the change in the output is not directly proportional to the change in the input.^18^ This is achieved by calculating and comparing the left and right sides of the conditions of the superposition principle (additivity and homogeneity). The ${\mathrm{NLD}}_{\text{object}}$ image is the residual of a comparison between the average of the acquired image data before and after reconstruction. An ${\mathrm{NLD}}_{\text{noise}}$ image is the residual of a comparison between noise data reconstructed with and without an object being present. The ${\mathrm{NLD}}_{\text{object}}$ image thus describes the systematic ${\mathrm{NLD}}_{\text{object}}$, whereas the ${\mathrm{NLD}}_{\text{noise}}$ series represent a series of images describing the ${\mathrm{NLD}}_{\text{noise}}$. Both image types are presented in the image domain. For a linear reconstruction system, the residual is close to zero. However, the residual may visualize distortions originating from nonlinearities in the CT configuration, e.g., the detector response.

#### 2.1.1 Nonlinear distortion of objects

**Fig. 1:**
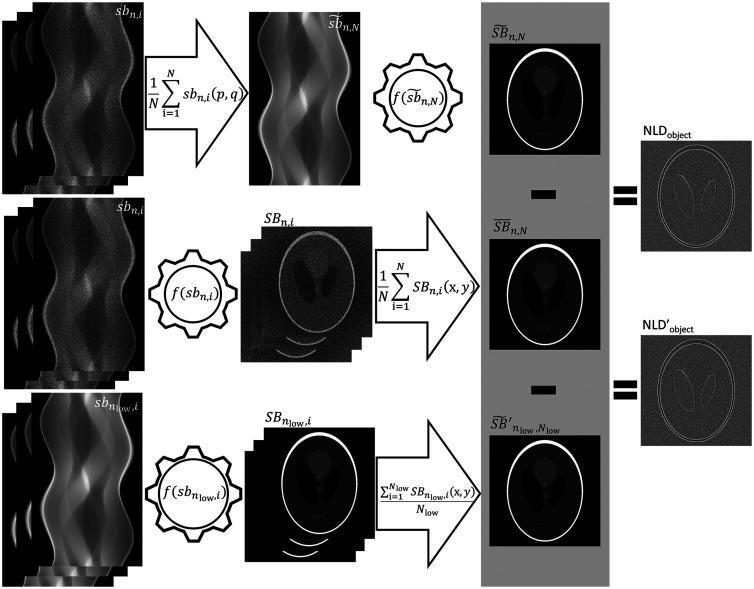
Workflow showing the calculation of the ${\mathrm{NLD}}_{\text{object}}$ and the ${\mathrm{NLD}}_{\text{object}}^{\prime }$. The arrows and cogwheels indicate averaging ($\frac{1}{N}\sum_{i=1}^{N}$) and reconstruction (f), respectively. The index i represents the acquisition number, and (x,y) and (p,q) indicate that averaging was performed in the image domain (middle and bottom rows of images) and the sinogram domain (top row of images), respectively. A series of N acquired noisy sinograms of the object ($sb_{n}$) was duplicated and averaged after reconstruction (i.e., a pixelwise average of the reconstructed images at the Cartesian coordinates x and y, middle row) and before reconstruction [i.e., a pixelwise average of the sinograms at the projection angles (p) and detector position (q), top row] to provide the ${\mathrm{NLD}}_{\text{object}}$ image that is the residual of these two calculations. The ${\mathrm{NLD}}_{\text{object}}^{\prime }$ was the residual of the reconstructed noisy sinograms (middle row) and the reconstructed low-noise sinograms indicated by the subscript low (bottom row). In the case of a linear reconstruction, the pixel values of the ${\mathrm{NLD}}_{\text{object}}$ image will be close to zero (i.e., an overall gray image).

A nonlinear noise reduction algorithm may distort the reproduction of an object in an image depending on the level of noise in the input data. For a CT system Ƥ, the input data are represented by a sinogram of an object s acquired with a background b and are written as

$$b_{n}(p,q)=Ƥ(s+b_{n})(p,q),$$

where the subscript n indicates the noise level of the background and p and q are the projection angle and detector position, respectively. Noise reduction in a CT system may be implemented during the reconstruction step. Hence, to isolate the nonlinear distortion introduced by a noise reduction algorithm, two reconstructions may be performed on a sinogram of the same object at different noise levels. One of the noise levels can be approximated to zero (noise-free) to obtain as large a difference in the comparison of the distortion as possible (the distortion is assumed to be smaller at lower noise levels). If manipulation of the sinogram is possible, the acquisition of a sinogram at a background n ($sb_{n}$) can be repeated N times and averaged before reconstruction to estimate the noise-free sinogram ${\overset{˜}{sb}}_{n,N}$ (provided N is sufficiently large). The reconstruction of this sinogram represents the object with the least nonlinear distortion ${\overset{˜}{SB}}_{n,N}$. In images reconstructed at high noise levels, there is a risk that the distortion of the object may be obscured by the distortion of the high noise level. It may therefore be more appropriate to consider the systematic nonlinear distortion. Thus the acquired sinograms $sb_{n}$ should be reconstructed separately and then averaged after reconstruction to represent both the reconstructed object and the systematic nonlinear distortion. Any difference between this averaged image (${\overset{¯}{SB}}_{n,N}$) and the image reconstructed from the approximate noise-free sinogram (${\overset{˜}{SB}}_{n,N}$) will result in a residual consisting of the systematic nonlinear distortion of the object and is written as

$${\mathrm{NLD}}_{\text{object}}={\overset{¯}{SB}}_{n,N}(x,y)-{\overset{˜}{SB}}_{n,N}(x,y),$$ (1)

where x and y are the reconstructed pixel positions in Cartesian coordinates (Fig. 1, workflow of the calculation of the ${\mathrm{NLD}}_{\text{object}}$ image, the top and middle rows of the images). When manipulation of the sinograms is not possible, an approximate noise-free image of the object ${\overset{˜}{SB}}_{n_{\text{low}},N_{\text{low}}}^{\prime }$ acquired separately may be compared with the average of the high-noise images (${\overset{¯}{SB}}_{n,N}$) to estimate an approximation of the ${\mathrm{NLD}}_{\text{object}}$ image, and this is written as

$${\mathrm{NLD}}_{\text{object}}^{\prime }={\overset{¯}{SB}}_{n,N}(x,y)-{\overset{˜}{SB}}_{n_{\text{low}},N_{\text{low}}}^{\prime }(x,y),$$ (2)

where the subscripts $n_{\text{low}}$ and $N_{\text{low}}$ indicate the noise level of the background and the number of repeated acquisitions for the approximate noise-free estimation, respectively (Fig. 1 gives a comparison of the workflow for the calculation of the ${\mathrm{NLD}}_{\text{object}}$ image and ${\mathrm{NLD}}_{\text{object}}^{\prime }$, the middle and bottom rows of images, respectively). Each of these images (${\mathrm{NLD}}_{\text{object}}$ and ${\mathrm{NLD}}_{\text{object}}^{\prime }$) provides a map of the nonlinear distortion that characterizes the systematic nonlinear distortion of the reconstructed object. However, the noise in these images does not have the same origin, and the random uncertainty in the resulting systematic nonlinear distortion will be higher than in the case in which sinogram manipulation is possible.In the application of the proposed method, using simulations of a CT acquisition of a typical abdominal image (see Sec. 2.2 for details), the acquisition of $sb_{n}$ was repeated 16, 32, 64, 128, and 256 times (N=16, 32, 64, 128, and 256) to illustrate how the difference in the uncertainty between the ${\mathrm{NLD}}_{\text{object}}$ and ${\mathrm{NLD}}_{\text{object}}^{\prime }$ changes with the number of repetitions. For the case in which manipulation of the sinograms was possible, these sinograms were averaged to give an approximate noise-free sinogram:

$${\overset{˜}{sb}}_{n,N}(p,q)=\frac{1}{N}\overset{N}{\underset{i=1}{\sum }}sb_{n,i}(p,q),$$ (3)

where index i is the acquisition number and N is the number of acquisitions at background noise level $b_{n}$ (p and q are still the projection angle and the detector position, respectively, Fig. 1). The reconstruction of ${\tilde{sb}}_{n,N}$ provides an estimate of the noise-free image of the object and was calculated according to

$${\overset{˜}{SB}}_{n,N}(x,y)=f({\overset{˜}{sb}}_{n,N}(p,q))(x,y),$$ (4)

where f is the reconstruction algorithm and x and y are the reconstructed pixel positions in Cartesian coordinates. The averaged image of the N acquired and reconstructed images at background noise level $b_{n}$ was calculated from

$${\overset{¯}{SB}}_{n,N}(x,y)=\frac{1}{N}\overset{N}{\underset{i=1}{\sum }}f(sb_{n,i}(p,q))(x,y).$$ (5)

The ${\mathrm{NLD}}_{\text{object}}$ image was calculated by inserting Eqs. (4) and (5) into Eq. (1) (Fig. 1) at background noise level $b_{n}$. The ${\overset{˜}{SB}}_{n_{\text{low}},N_{\text{low}}}^{\prime }$ in the calculation of the ${\mathrm{NLD}}_{\text{object}}^{\prime }$ image in Eq. (2) can, if the acquisition procedure change nonlinearly due to for example change in size of the x-ray focal spot as the tube current is increased, be estimated by an average of many reconstructed images of the object such as the ${\overset{¯}{SB}}_{n,N}$ [Eq. (5)]. However, this study used simulations of CT acquisitions with fixed acquisition parameters to focus on the nonlinearities in the reconstruction algorithm. Hence, the ${\overset{˜}{SB}}_{n_{\text{low}},N_{\text{low}}}^{\prime }$ was estimated using only one separate acquired image with a noise level corresponding to the average of the high-noise images (${\overset{¯}{SB}}_{n,N}$). The noise level was defined at a contrast-to-noise ratio (CNR) dependent on the simulated noise level and the number of repeated acquisitions.

#### 2.1.2 Nonlinear distortion of noise

**Fig. 2:**
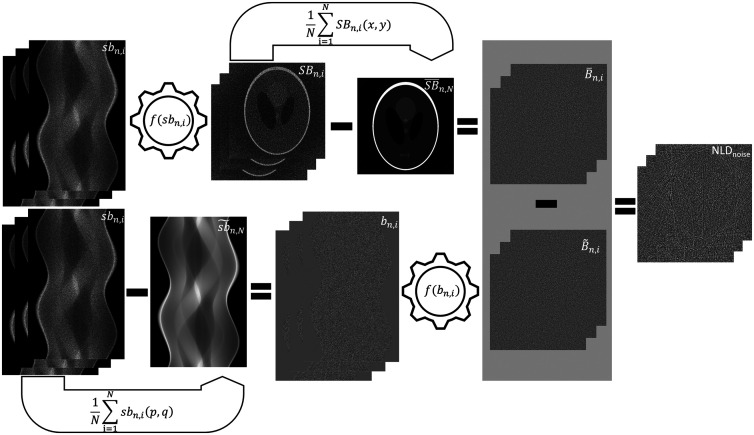
Workflow showing the calculation of the systematic ${\mathrm{NLD}}_{\text{noise}}$. Arrows and cogwheels indicate averaging ($\frac{1}{N}\sum_{i=1}^{N}$) and reconstruction (f), respectively. The index i is the acquisition number, and (x,y) and (p,q) indicate that averaging was performed in the image domain and the sinogram domain, respectively. A series of N sinograms of an object s acquired at noise level n ($sb_{n}$) was duplicated. The first series was used to obtain the isolated noise images (with noise level n) reconstructed in the presence of the object (${\overset{¯}{B}}_{n,i}$, top row), i.e., the mean of the reconstructed noisy sinograms (${\overset{¯}{SB}}_{n,N}$) was reconstructed and subtracted from each reconstructed noisy image of the object ($SB_{n,i}$). The second series was used to obtain the isolated noise images (with noise level n) reconstructed in the absence of the object (${\tilde{B}}_{n,i}$, bottom row), i.e., the mean of the acquired noisy sinograms (${\tilde{sb}}_{n,N}$) was subtracted from each acquired noisy sinogram of the object ($sb_{n,i}$) and then reconstructed. Finally, the series of ${\mathrm{NLD}}_{\text{noise}}$ images was obtained by subtracting each of the isolated noise images reconstructed in the absence of the object (${\tilde{B}}_{n,i}$) from each of the isolated noise images reconstructed in the presence of the object (${\overset{¯}{B}}_{n,i}$). In the case of a linear reconstruction, the pixel values of the ${\mathrm{NLD}}_{\text{noise}}$ image would be close to zero.

When the noise reduction algorithms are nonlinear, the distortion may be different at each acquisition as quantum noise is generated randomly. Further, the noise reduction algorithm may reconstruct noise differently when an object is present. It is then possible to estimate the ${\mathrm{NLD}}_{\text{noise}}$ by comparing the noise reconstructed with and without an object being present. However, the noise must be isolated from the object in both cases. The noise in an image reconstructed with the object being present can be isolated after reconstruction by subtracting an estimate of the reconstructed object S^(x,y) from each noisy image of the object $SB_{n,i}$. In the case in which the noise is reconstructed without an object being present, the noise is isolated before reconstruction by subtracting an estimate of the object sinogram s^(p,q) from each noisy sinogram $sb_{n,i}$. Thus the ${\mathrm{NLD}}_{\text{noise}}$ for a series of images is the difference between each pair of isolated noise images, which is written as

$${\mathrm{NLD}}_{\text{noise}}={\overset{¯}{B}}_{n,i}(x,y)-{\overset{˜}{B}}_{n,i}(x,y),$$ (6)

where ${\overset{¯}{B}}_{n,i}$ and ${\tilde{B}}_{n,i}$ are the noise images reconstructed with and without the object being present, respectively; i has values from 1 to N and represents the acquisition number of the reconstructed noise; and n denotes the level of noise at which the ${\mathrm{NLD}}_{\text{noise}}$ series is assessed (Fig. 2, workflow of the calculation of the ${\mathrm{NLD}}_{\text{noise}}$ series). Each of the images in the ${\mathrm{NLD}}_{\text{noise}}$ series provides a map of the nonlinear distortion that characterizes the random nonlinear distortion of the reduced noise. An assessment using a linear reconstruction algorithm will result in an ${\mathrm{NLD}}_{\text{noise}}$ series with pixel values close to zero.

In the application of the proposed method (see Sec. 2.2), the average of the acquired noisy images ${\overset{¯}{SB}}_{n,N}$ [described in Eq. (5)] was used as the estimate of the reconstructed object (S^(x,y)), and the calculation of ${\overset{¯}{B}}_{n,i}$ was expressed as

$${\overset{¯}{B}}_{n,i}(x,y)=SB_{n,i}(x,y)-{\overset{¯}{SB}}_{n,N}(x,y),$$

where $SB_{n,i}$ is the i’th reconstructed noisy image of the object at noise level n (Fig. 2). The average of the acquired noisy sinograms ${\overset{˜}{sb}}_{n,N}$ [described by Eq. (3)] was used as the estimate of the object sinogram, and the calculation of ${\tilde{B}}_{n,i}$ was expressed as

$${\overset{˜}{B}}_{n,i}(x,y)=f(sb_{n,i}(p,q)-{\overset{˜}{sb}}_{n,N}(p,q))(x,y),$$

where f is the reconstruction algorithm and $sb_{n,i}$ is the i’th noisy sinogram of the object at noise level n (Fig. 2). The ${\mathrm{NLD}}_{\text{noise}}$ series was calculated for the same number of repeated acquisitions used for the ${\mathrm{NLD}}_{\text{object}}$ images (N=16, 32, 64, 128, and 256), using Eq. (6). However, for the estimation of the ${\mathrm{NLD}}_{\text{noise}}$ series in this study, N was equal to 256 to show the ${\mathrm{NLD}}_{\text{noise}}$ series with negligible uncertainties. Further, the ${\mathrm{NLD}}_{\text{noise}}$ series consisted of N separate estimates of the ${\mathrm{NLD}}_{\text{noise}}$.

### 2.2 Application of the Method

As described above, the method was evaluated using simulations. The open-source Compute Unified Device Architecture-integrated CT simulation toolbox [ASTRA© (version 1.9.9.dev1)^19^^–^^21^ for MATLAB™ (R2020b)] was used to perform reconstructions of the test object. In the toolbox, the projection geometry was set to fan-beam using a flat array of the detector. The 1474 detector elements with a detector element width of 1 mm (defined at the isocenter) were used to reconstruct images with a volume of 1×768×768  voxels with equilateral voxel size of $1\;{\mathrm{mm}}^{3}$. However, the distortion was assessed in the trans-axial image plane using a 512×512  pixel region of interest (ROI) to represent the most common field of view used clinically. The 1152 projection angles were evenly spaced around a whole rotation. The source-to-detector distance was set to 1085.6 mm, and the isocenter was placed 696.7 mm from the source. A function incorporated in the toolbox was used to compute forward projections of the test object (in this case representing a sinogram map of linear attenuation) with respect to the volume and projection geometry.

**Fig. 3:**
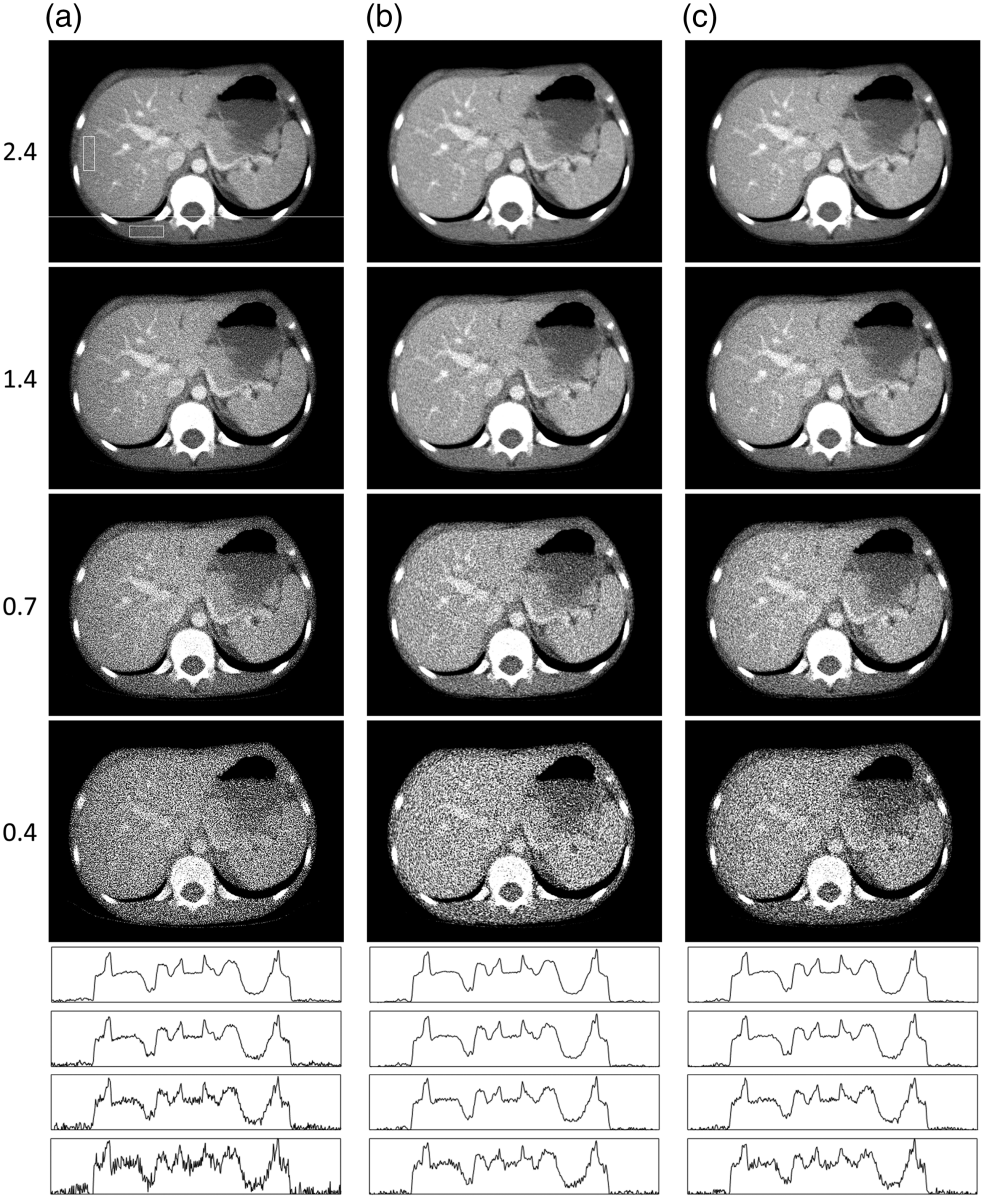
Reconstructed image of a typical abdominal CT image at CNRs of 2.4 (top row), 1.4, 0.7, and 0.4 (bottom row) for (a) FBP alone and the two combinations of reconstruction algorithms and noise reduction algorithms described in the text. (b) ${\mathrm{SIRT}}_{\mathrm{med}}$ and (c) ${\mathrm{CGLS}}_{\mathrm{TV}}$. Horizontal profile plots positioned in each image [as shown by the horizontal white line in the FBP image for a CNR of 2.4, in (a)] are shown below the images to better illustrate the changes in the magnitude of the noise. The profile plots are arranged by CNR from the highest (2.4: top) to the lowest (0.4: bottom) for each image (a)–(c).

Poisson noise was used to simulate noise originating from the simulated CT acquisition of the test object. The CT simulation generated a sinogram mapping the linear attenuation that was first transformed by Beer–Lambert’s law into the detector signal, where the noise was added and then transformed back into linear attenuation data. The final sinograms used in the calculation of the ${\mathrm{NLD}}_{\text{object}}$ and ${\mathrm{NLD}}_{\text{noise}}$ images may be averaged both at the level of detector signal or at the attenuation data. The averaging in this study was done on the attenuation data as the noise reduction algorithms investigated were only working on the attenuation data and did not involve transformation from the detector signal. A CNR was defined in an FBP-reconstructed image as the difference between the CT number of liver and muscle tissue divided by the standard deviation of the CT number for the muscle tissue. The estimation of the CT number of the tissues was determined using ROIs sufficiently large to generate stable standard deviations [In Fig. 3(a), the location of the ROIs is indicated in the FBP image of CNR=2.4]. The expected value in the Poisson noise calculation was varied so that the linear attenuation coefficient ranged between that for dry air (at near sea level) and cortical bone at 60 keV, taken from the NIST database.^22^ CNR values of 0.4, 0.7, 1.4, and 2.4 were used to determine the nonlinear performance of the noise reduction algorithms at different noise levels.

Two combinations of the nonlinear noise reduction algorithm and reconstruction algorithm were tested: a median filter (using a kernel of 3×3) in combination with the simultaneous iterative reconstruction technique (SIRT, ${\mathrm{SIRT}}_{\mathrm{med}}$) and a total variation algorithm^23^ (TV-L1,^24^^,^^25^ henceforth denoted TV) in combination with the conjugate gradient least-squares algorithm (CGLS, ${\mathrm{CGLS}}_{\mathrm{TV}}$). Each nonlinear noise reduction algorithm was applied to the sinograms before reconstruction. Both iterative reconstruction algorithms were stopped after 100 iterations, and an image reconstructed with FBP in combination with each respective nonlinear noise reduction algorithm (using a Hamming filter) was used as the initial estimate in the iteration sequence. An image with high noise has high total variation, which is the integral of the absolute image gradient. A TV denoising algorithm can reduce noise by minimizing the total variation while preserving the edges of objects in an image as noise contributes more to the total variation of the image than edges. The amount of denoising of the algorithm was adjusted by the regularization parameter λ, where a too high λ could lead to blurring of edges as the parameter allows the image to be less consistent with the noisy image. The denoised image was estimated iteratively, and the number of iterations $N_{\mathrm{iter}}$ and the amount of denoising λ were set to 50 and 1.9, respectively, to match the noise reduction (the reduction of standard deviation) of a median filter. Further, to obtain equal contrast after processing with the TV algorithm, the mean ratio between unprocessed and processed sinograms was calculated to allow for correction of the contrast level due to the normalization process in the TV algorithm.

The visualization of the average nonlinear distortion could not show the distribution of the distortion. However, each repeated acquisition was an estimation of the nonlinear distortion. Hence, for each investigated algorithm, a pixelwise distribution of the distortion was estimated as an ${\mathrm{NLD}}_{\text{object}}$ image at the 5^th^, 50^th^, and 95^th^ percentiles together with a plot of the corresponding horizontal profile [In Fig. 3(a), the location of the horizontal profile plots is indicated in the FBP image of CNR=2.4]. The distribution of the nonlinear distortion was analyzed in more detail by depicting the sharp edge of the dexter costa against the lung (the more peripherally located costa, left in the image) using 20 pixels at the same horizontal profile as described before. Plots of both the ${\mathrm{NLD}}_{\text{object}}$, the 5^th^, 50^th^, and 95^th^ percentiles together with the profiles of the ${\tilde{SB}}_{n,N}$ and the ${\overset{¯}{SB}}_{n,N}$ were viewed to illustrate the correlation between the object and nonlinear distortion.

## 3 Results

**Fig. 4:**
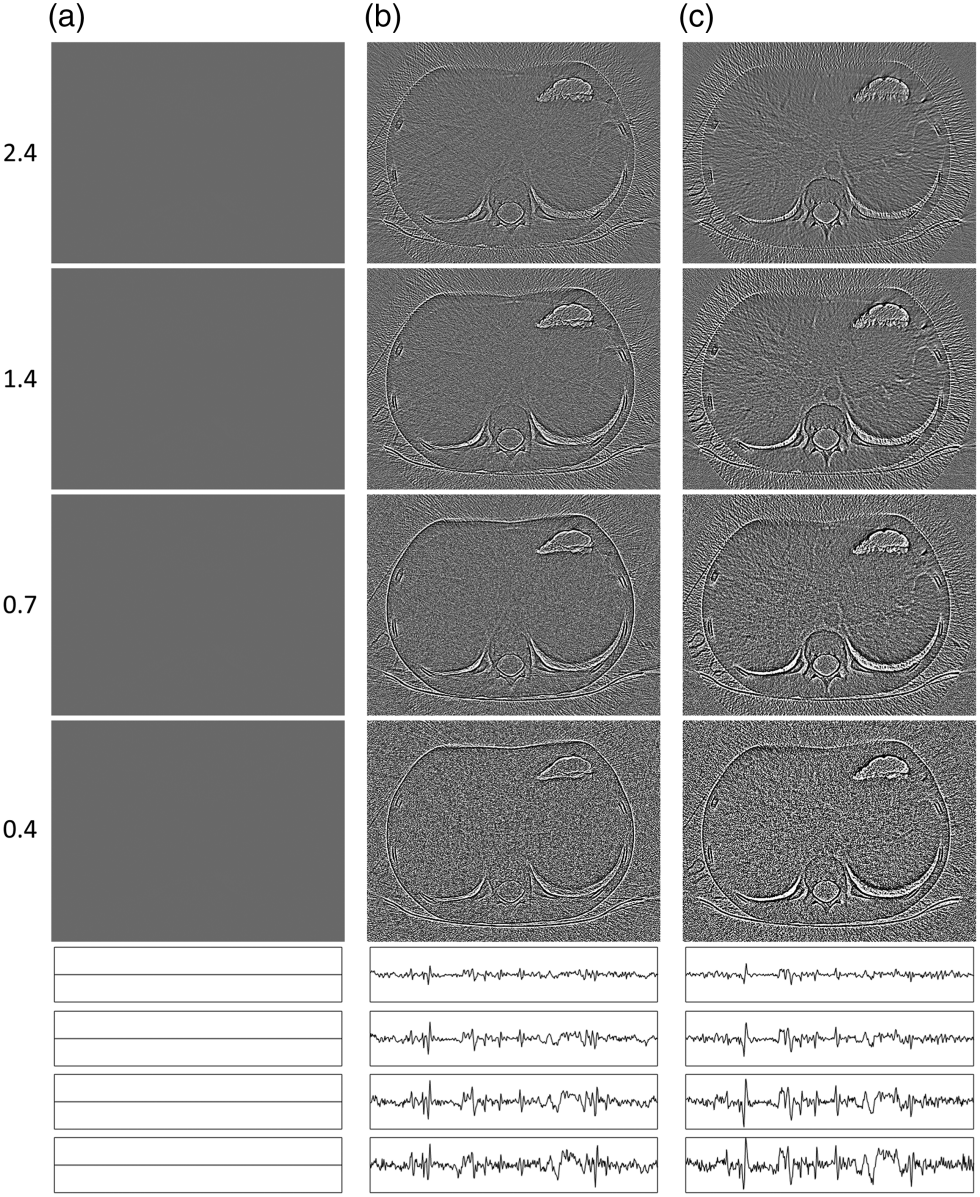
Nonlinear distortion images of objects (${\mathrm{NLD}}_{\text{object}}$, window −10 to 10 HU) at CNRs of 2.4 (top row), 1.4, 0.7, and 0.4 (bottom row) estimated using 256 repeated acquisitions for (a) FBP alone and the two combinations of reconstruction algorithms and noise reduction algorithms described in the text. (b) ${\mathrm{SIRT}}_{\mathrm{med}}$ and (c) ${\mathrm{CGLS}}_{\mathrm{TV}}$. Horizontal profile plots positioned in each ${\mathrm{NLD}}_{\text{object}}$ image [as shown by the horizontal white line in the FBP image for a CNR of 2.4, in Fig. 3(a)] are shown below the images to better illustrate the changes in the magnitude of the distortion (amplitude range: −70 to 70 HU). The profile plots are arranged by CNR from the highest (2.4: top) to the lowest (0.4: bottom) for each image (a)–(c).

The noise reduction, in terms of the standard deviation (σ), was similar for both combinations of the reconstruction algorithm and noise reduction algorithm (${\mathrm{SIRT}}_{\mathrm{med}}$ and ${\mathrm{CGLS}}_{\mathrm{TV}}$) at the three lowest CNRs (Table 1 and Fig. 3). At these CNRs, each combination reduced the standard deviation by ∼40% in the ROI in the longissimus muscle (Table 1, indicated by the white square in the muscle Fig. 3(a) at CNR=2.4) and had a smoothing effect on the noise texture (Fig. 3). The noise reduction of ${\mathrm{SIRT}}_{\mathrm{med}}$ was slightly stronger than the other combination (Table 1). The nonlinear distortion of FBP [Figs. 4(a) and 6(a)] was negligible, and the maximum deviation from zero in any of the images of the nonlinear distortion (${\mathrm{NLD}}_{\text{object}}$ and ${\mathrm{NLD}}_{\text{noise}}$) was in the range of $\pm 10^{-3}\;\mathrm{HU}$.

**Table 1:** Noise at four CNRs, in terms of standard deviation (σ) in a 60×20  pixel ROI in the longissimus muscle [the location of the ROI is shown in the FBP image of CNR=2.4, in Fig. 3(a)] for FBP alone and two combinations of reconstruction algorithms and noise reduction algorithms described in the text (${\mathrm{SIRT}}_{\mathrm{med}}$ and ${\mathrm{CGLS}}_{\mathrm{TV}}$).

CNR	σ FBP^a^	σ ${\mathrm{SIRT}}_{\mathrm{med}}$ ^b^	σ ${\mathrm{CGLS}}_{\mathrm{TV}}$ ^c^
2.4	23	15	19
1.4	41	24	27
0.7	79	43	48
0.4	157	87	85

a σ FBP, standard deviation of the filtered back-projection.

b σ ${\mathrm{SIRT}}_{\mathrm{med}}$, standard deviation of the simultaneous iterative reconstruction technique with a median filter.

c σ ${\mathrm{CGLS}}_{\mathrm{TV}}$, standard deviation of the conjugate gradient least-squares algorithm with a total variation algorithm.

### 3.1 Nonlinear Distortion of Objects

**Fig. 5:**
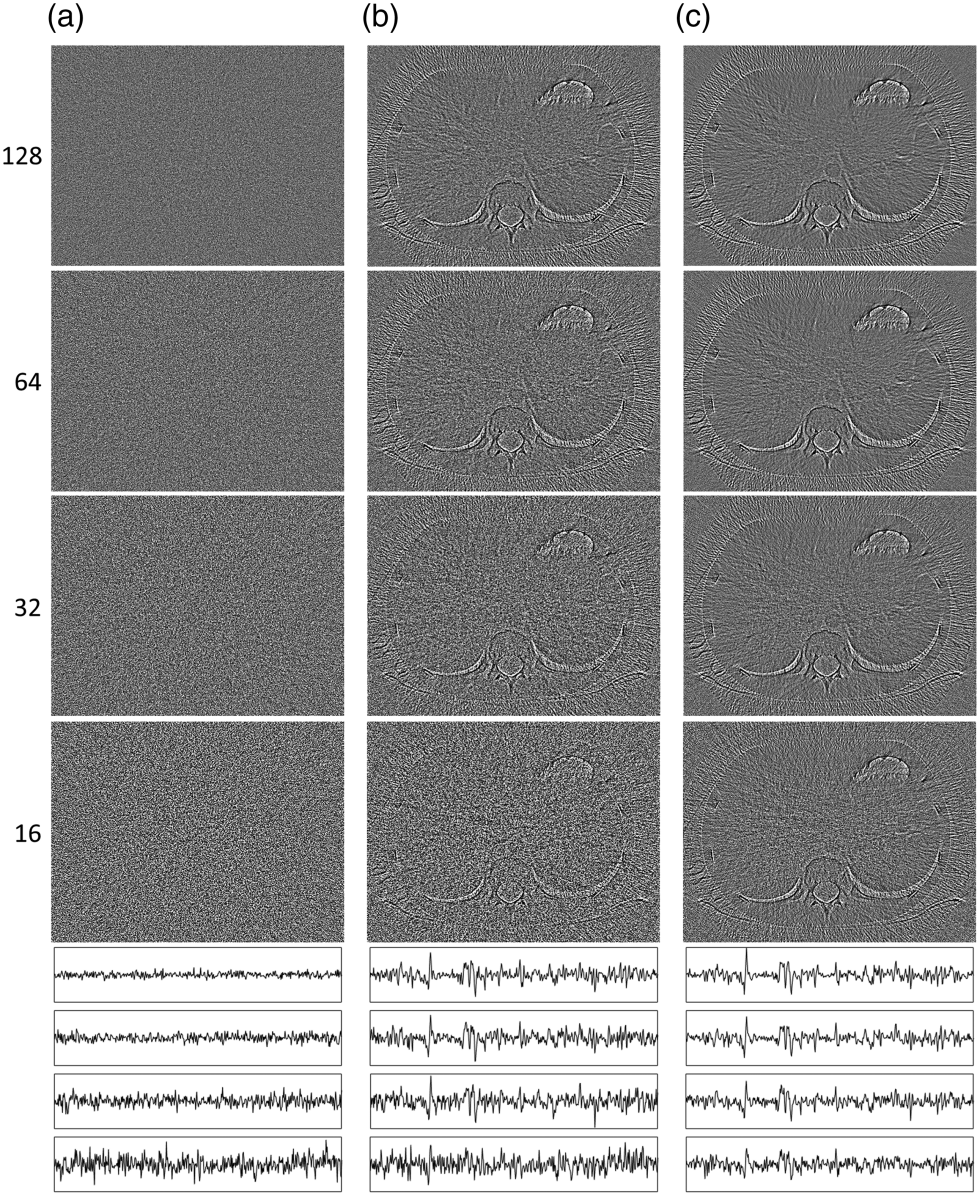
Approximation of the nonlinear distortion images of objects (${\mathrm{NLD}}_{\text{object}}^{\prime }$, window −10 to 10 HU) at CNRs of 2.4 estimated using 128 (top row), 64, 32, and 16 (bottom row) repeated acquisitions for (a) FBP alone and the combination of the reconstruction algorithm and noise reduction algorithm and (b) ${\mathrm{CGLS}}_{\mathrm{TV}}$, described in the text. (c) The ${\mathrm{NLD}}_{\text{object}}$ for ${\mathrm{CGLS}}_{\mathrm{TV}}$ is estimated using the same number of repetitions as for the ${\mathrm{NLD}}_{\text{object}}^{\prime }$ for comparison. Horizontal profile plots positioned in each ${\mathrm{NLD}}_{\text{object}}^{\prime }$ image [as shown by the horizontal white line in the FBP image for a CNR of 2.4, in Fig. 3(a)] are shown below the images to better illustrate the changes in the magnitude of the distortion (amplitude range: −30 to 30 HU). The profile plots are arranged by the number of repeated acquisitions from the highest (128: top) to the lowest (16: bottom) for each image (a)–(c).

The ${\mathrm{NLD}}_{\text{object}}$ image was used to identify the nonlinear properties of an algorithm by visualizing the systematic nonlinear distortion in the reconstruction of objects at a given CNR. As expected, FBP did not show any nonlinear distortion [Fig. 4(a)]. For the two combinations investigated, the ${\mathrm{NLD}}_{\text{object}}$ images showed distortions at most edges of the anatomical structures in the CT image used to test the method (Figs. 4(b) and 4(c)]. These structures of distortion in the ${\mathrm{NLD}}_{\text{object}}$ image indicate smoothing of the reconstructed object caused by the nonlinear algorithm as the noise increases. The ${\mathrm{NLD}}_{\text{object}}$ image of ${\mathrm{SIRT}}_{\text{med}}$ was indicated to cause less systematic distortion than the ${\mathrm{CGLS}}_{\mathrm{TV}}$ algorithm [Figs. 4(b) and 4(c)]. One indication was the more prominent distortion at the contrast filled vessels in the ${\mathrm{NLD}}_{\text{object}}$ image of ${\mathrm{CGLS}}_{\mathrm{TV}}$ [Fig. 4(c)]. A dark region in the ${\mathrm{NLD}}_{\text{object}}$ images indicates a nonlinear contrast reduction at the CNR tested. Compared with using the ${\mathrm{SIRT}}_{\mathrm{med}}$ algorithm, the contrast reduction was higher using the ${\mathrm{CGLS}}_{\mathrm{TV}}$ algorithm, especially for the contrast filled vessels [Fig. 4(c)]. Both algorithm combinations exhibited a CNR dependence such that the edge distortions were more pronounced at lower CNRs [Figs. 4(b) and 4(c)]. Between the two combinations investigated, ${\mathrm{CGLS}}_{\mathrm{TV}}$ showed the strongest CNR dependence in both edge distortion and contrast reduction [Fig. 4(c)]. The random uncertainty in the ${\mathrm{NLD}}_{\text{object}}$ image was higher at lower CNRs due to the higher noise (Fig. 4). A comparison between the ${\mathrm{NLD}}_{\text{object}}$ image and its approximation, the ${\mathrm{NLD}}_{\text{object}}^{\prime }$ image, indicated good correspondence other than additional noise (higher random uncertainty) in the ${\mathrm{NLD}}_{\text{object}}^{\prime }$ image (Fig. 5). The uncertainty in both ${\mathrm{NLD}}_{\text{object}}$ and ${\mathrm{NLD}}_{\text{object}}^{\prime }$ was increased as the number of repeated acquisitions was decreased (Fig. 5). However, the estimation of the ${\mathrm{NLD}}_{\text{object}}$ by the ${\mathrm{NLD}}_{\text{object}}^{\prime }$ was shown to be comparable at 128 repetitions, where most of the distorted structures could be detected [Fig. 5(b) and 5(c)]. The estimation of the ${\mathrm{NLD}}_{\text{object}}$ itself was almost unchanged down to between 32 and 64 repetitions [Fig. 5(c)].

**Fig. 6:**
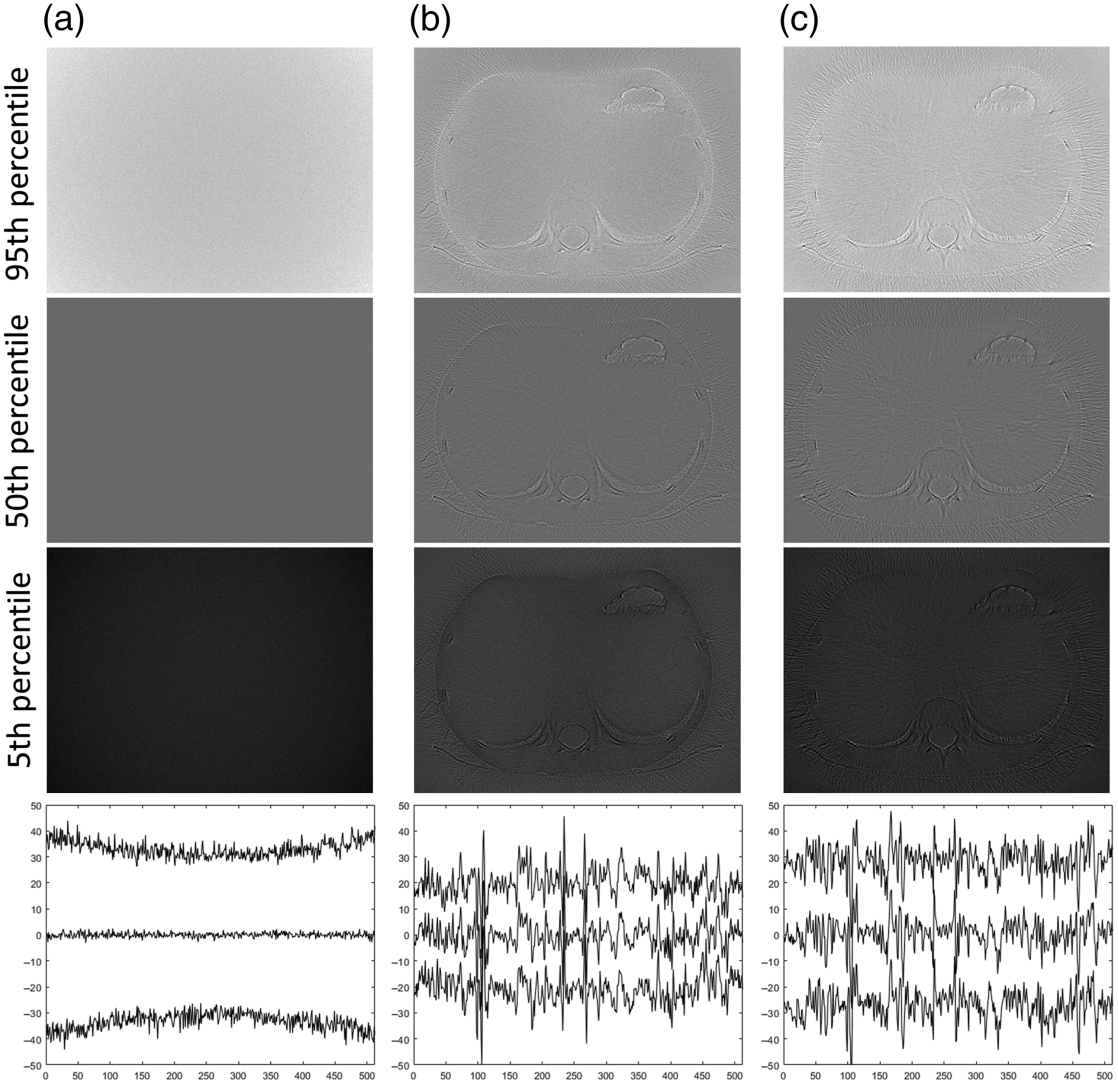
The 95th (top row), 50th (middle row), and 5th (bottom row) percentile representations of the nonlinear distortion images of objects (${\mathrm{NLD}}_{\text{object}}$, window −50 to 50 HU) at a CNR of 2.4 estimated using 256 repeated acquisitions for (a) FBP alone and the two combinations of reconstruction algorithms and noise reduction algorithms described in the text. (b) ${\mathrm{SIRT}}_{\mathrm{med}}$ and (c) ${\mathrm{CGLS}}_{\mathrm{TV}}$. Horizontal profile plots positioned in each percentile image [as shown by the horizontal white line in the FBP image for a CNR of 2.4, in Fig. 3(a)] are shown below the images to better illustrate the changes in the magnitude of the distortion (amplitude range: −50 to 50 HU). The profile plots are arranged by the percentile level from the highest (95th: top) to the lowest (5th: bottom) for each image (a)–(c).

The distribution of the distortion visualized by the percentile images (5th and 95th) showed the distribution of ${\mathrm{NLD}}_{\text{object}}$ of the investigated algorithm to have a maximum range between about −40 to 40 HU (Fig. 6). Further, the variation of the distortion across space was small between the percentile images (Fig. 6). A comparison between the distribution estimation at the 5th- and 95th-percentile plots showed the nonlinear distortion to have a smaller amplitude in general for the noise reduction algorithms (${\mathrm{SIRT}}_{\mathrm{med}}$ and ${\mathrm{CGLS}}_{\mathrm{TV}}$) than the noise for FBP (not affected by nonlinear distortion, Fig. 6). The general amplitude level of the 5th and 95th percentiles for the ${\mathrm{SIRT}}_{\mathrm{med}}$, ${\mathrm{CGLS}}_{\mathrm{TV}}$, and FBP was about −20 and 20 HU, −30 and 30 HU, and −40 and 40 HU, respectively (Fig. 6). However, the nonlinear distortion had amplitude peaks in the profile higher than the noise distribution of FBP. The distribution in space at the 50th-percentile plot for FBP was shown to be noisier than the ${\mathrm{NLD}}_{\text{object}}$ for the same algorithm [compare the profile plots in Figs. 4(a) and 6(a)].

**Fig. 7:**
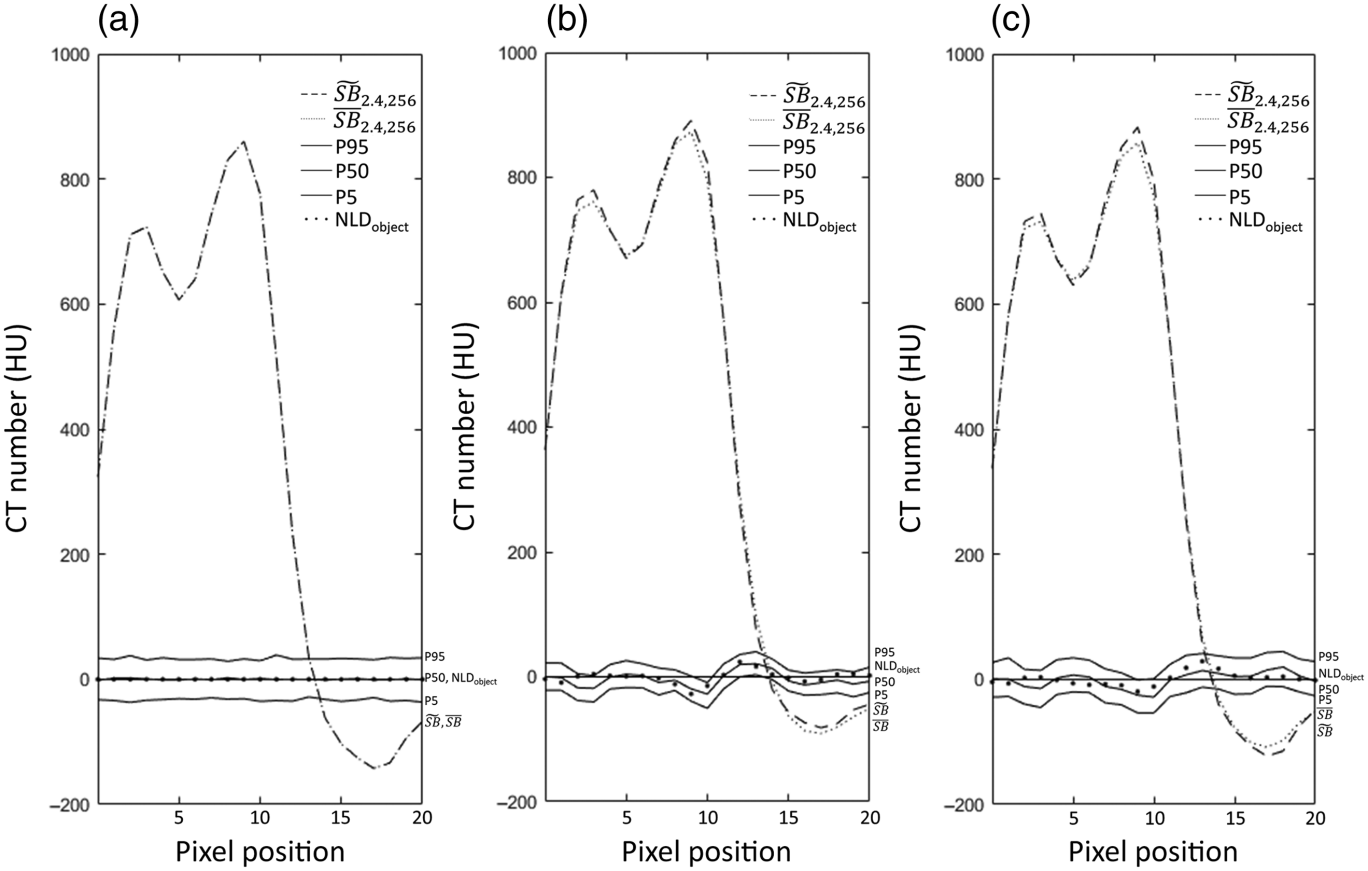
Detailed analysis of the sharp edge of a costa against the lung [using a profile of 20 pixels, as shown by the horizontal white line in the FBP image for a CNR of 2.4, in Fig. 3(a)] illustrating the correlation in space between the position of the edge (edge reconstructed in noise-free, ${\tilde{SB}}_{2.4,256}$, dashed line, versus high noise, ${\overset{¯}{SB}}_{2.4,256}$, thin dotted line) and the nonlinear distortion (${\mathrm{NLD}}_{\text{object}}$, thick dotted line). The distortion distribution was illustrated using the 95th (top solid line), 50th (middle solid line), and 5th percentile (bottom solid line). The analysis was performed at a CNR of 2.4 using 256 repeated acquisitions for (a) FBP alone and the two combinations of reconstruction algorithms and noise reduction algorithms described in the text. (b) ${\mathrm{SIRT}}_{\mathrm{med}}$ and (c) ${\mathrm{CGLS}}_{\mathrm{TV}}$.

The more detailed analysis of the sharp edge of a costa against the lung illustrated the correlation in space between the ${\mathrm{NLD}}_{\text{object}}$ and the variations of ${\tilde{SB}}_{2.4,256}$ and ${\overset{¯}{SB}}_{2.4,256}$ for the investigated algorithms (Fig. 7). The FBP algorithm did not show any distortion, which was shown by the flat line of the ${\mathrm{NLD}}_{\text{object}}$ and the overlapping lines of ${\tilde{SB}}_{2.4,256}$ and ${\overset{¯}{SB}}_{2.4,256}$ [Fig. 7(a)]. Further, the noise reduction algorithms (${\mathrm{SIRT}}_{\mathrm{med}}$ and ${\mathrm{CGLS}}_{\mathrm{TV}}$) showed specific characteristics of the nonlinear distortion; for example, the distortion at the lung had different signs for the two algorithms (pixel 15 to 20, Figs. 7(b) and 7(c)]. The distortion at the edge (pixel 10 to 15) for the noise reduction algorithms was visualized by the ${\mathrm{NLD}}_{\text{object}}$ even though the difference between ${\tilde{SB}}_{2.4,256}$ and ${\overset{¯}{SB}}_{2.4,256}$ was difficult to see. As expected, the estimation of the distortion distribution by the percentile profiles was shown to be affected by noise. Further, the profile of the 50th percentile and the ${\mathrm{NLD}}_{\text{object}}$ for each algorithm were shown to be equal. However, the amplitude of the distortion described by these profiles was shown to have a variation as large as the difference between the 5th and 95th percentiles.

### 3.2 Nonlinear Distortion of Noise

**Fig. 8:**
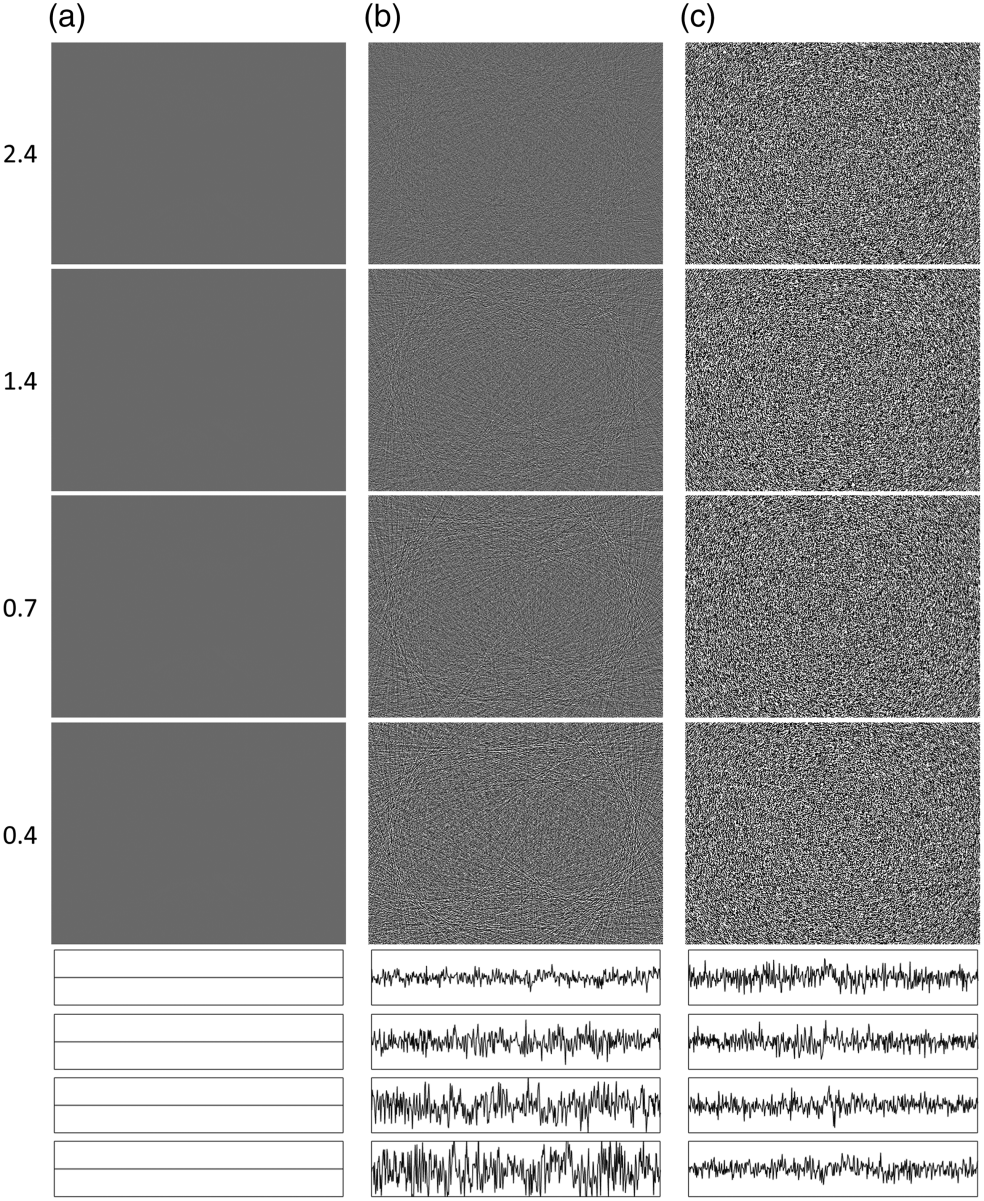
Nonlinear distortion images of noise (${\mathrm{NLD}}_{\text{noise}}$, one image of the series of images is shown, window −50 to 50 HU) at CNRs of 2.4 (top row), 1.4, 0.7, and 0.4 (bottom row) estimated using 256 repeated acquisitions for (a) FBP alone and the two combinations of reconstruction algorithms and noise reduction algorithms described in the text. (b) ${\mathrm{SIRT}}_{\mathrm{med}}$ and (c) ${\mathrm{CGLS}}_{\mathrm{TV}}$. Horizontal profile plots positioned in each ${\mathrm{NLD}}_{\text{noise}}$ image [as shown by the horizontal white line in the FBP image for a CNR of 2.4, in Fig. 3(a)] are shown below the images to better illustrate the changes in the magnitude of the distortion (amplitude range for FBP and ${\mathrm{SIRT}}_{\mathrm{med}}$, −50 to 50 HU and for ${\mathrm{CGLS}}_{\mathrm{TV}}$, −5000 to 5000 HU). Note: the amplitude range has been increased by a factor 100 for ${\mathrm{CGLS}}_{\mathrm{TV}}$ to better visualize the differences in magnitude between the CNRs. The profile plots are arranged by CNR from the highest (2.4: top) to the lowest (0.4: bottom) for each image (a)–(c).

The ${\mathrm{NLD}}_{\text{noise}}$ series (Fig. 8) shows the difference in nonlinear distortion between two images when reconstructing the same noise with and without an object being present. This allows the consistency in noise reduction and the dependence on the object to be visualized. The linear reconstruction algorithm FBP did not show any nonlinear distortion of the noise [Fig. 8(a)]. Among the two combinations investigated, ${\mathrm{SIRT}}_{\mathrm{med}}$ was the only one to show the structure of the distortion at contours of the anatomy in the abdominal CT image in the ${\mathrm{NLD}}_{\text{noise}}$ series [Fig. 8(b)]. However, the pixel values in the ${\mathrm{NLD}}_{\text{noise}}$ series obtained with ${\mathrm{SIRT}}_{\mathrm{med}}$ was about two orders of magnitude lower than for the ${\mathrm{CGLS}}_{\mathrm{TV}}$ combination [Figs. 8(b) and 8(c)]. The structures seemed to have a mean of zero as no structures were observed when the series of ${\mathrm{NLD}}_{\text{noise}}$ images was averaged (data not shown). The ${\mathrm{NLD}}_{\text{noise}}$ series showed a correlated noisy texture that varied depending on the combination. The magnitude of the pixel values in the ${\mathrm{NLD}}_{\text{noise}}$ series using the ${\mathrm{SIRT}}_{\mathrm{med}}$ combination was shown to increase as the CNR decreased such that the distorted structures became more distinct [Fig. 8(b)]. This is in contrast to the ${\mathrm{CGLS}}_{\mathrm{TV}}$ combination, which did not show any dependence on CNR [Fig. 8(c)].

## 4 Discussion

An ideal noise reduction algorithm decreases the noise power, preserves the object signal power, and does not cause any distortion. This paper presents two new types of images that were used to visualize the performance of a nonlinear noise reduction algorithm in terms of nonlinear distortion. The distortion of an image can be described in the frequency domain as the transfer of frequencies of an object to other frequencies, especially to harmonics of the object frequencies.^15^ However, in this study, the distortion is defined in the spatial domain as morphing of the image content, hence, including both object and noise. This is a broad definition of the distortion as it is more common to only estimate distortion when a severe morphing of the object has occurred. However, using the present definition, many of the effects of a nonlinear algorithm on the image quality can be understood to originate from distortion. One of the new types of images, the ${\mathrm{NLD}}_{\text{object}}$ image, visualized the systematic distortion by the difference between reconstructing an arbitrary object in high versus low noise; then it could indicate which structures were affected by the algorithm as the noise was increased. The noise power may also be distorted; hence, noise is also a part of the image content. Further, in the image domain, the noise may be distorted systematically such that noise mimics the object, as nonlinear noise reduction algorithms often depend on the object. The other type of image, the ${\mathrm{NLD}}_{\text{noise}}$ series, visualizes this distortion of noise by estimating the difference in reconstructing the noise with and without the object being present. Thus the NLD images did not test the algorithm against the ground truth, but to what extent the algorithm met the criteria for linear systems. Further, the test was based on comparing the average of many acquisition data before and after reconstruction, which should be equal for a linear system. Hence, the robustness of the algorithm was tested rather than the image quality. This concept of analyzing distortion was tested using a median filter in combination with the SIRT algorithm and a TV algorithm in combination with the CGLS algorithm.

An approximation of ${\mathrm{NLD}}_{\text{object}}$, ${\mathrm{NLD}}_{\text{object}}^{\prime }$, was also introduced based on assuming the distortion to be high compared with an averaged noise. By comparing one CT image acquired using a high dose and an average of many images acquired using a low dose, ${\mathrm{NLD}}_{\text{object}}^{\prime }$ demonstrates the systematic nonlinear distortion difference between these dose levels. Noise may interfere with the nonlinear distortion analysis if the high-dose image is not high enough. However, the acquisition of the high-dose image may also be repeated and averaged similar to the low-dose images to represent a CT acquisition of a higher dose and lower the random uncertainty of the distortion estimation. Further, the ${\mathrm{NLD}}_{\text{object}}^{\prime }$ image can be applied directly in an existing CT system to analyze the integrated noise reduction algorithms without the need to perform any mathematical operations on the raw data. However, it must be remembered that the nonlinear differences of an existing CT system acquisition configuration (e.g., size of the focal spot and number of acquired projections) between these dose levels could be visualized. Further, an ${\mathrm{NLD}}_{\text{object}}^{\prime }$ image may also visualize ring artifacts as the detector response between detector elements at a low dose may vary. This was not seen in this study as it was assumed in the simulations that the detectors had a perfect response. A more complex noise model including detector noise could perhaps have shown the effect of ring artifacts when simulating noise from CT examinations acquired at a low dose.^26^ Thus the degree of noise reduction by an algorithm would have showed to be limited by the quality and performance of the hardware components in the CT system. Further, these hardware effects may be isolated by analyzing a linear reconstruction algorithm, for example FBP, which will not exhibit nonlinear distortion effects due to the reconstruction algorithm.

In a dose optimization of CT examinations, an ${\mathrm{NLD}}_{\text{object}}^{\prime }$ image might be more useful than the ${\mathrm{NLD}}_{\text{object}}$ image as the ${\mathrm{NLD}}_{\text{object}}^{\prime }$ image accounts for all changes in the image quality between two dose levels. In contrast, ${\mathrm{NLD}}_{\text{object}}$ is more suited in an optimization procedure of a nonlinear noise reduction algorithm itself as it isolates the distortion of the nonlinear noise reduction algorithm. Further, a reasonable uncertainty in the ${\mathrm{NLD}}_{\text{object}}^{\prime }$ image was obtained at 128 repetitions of the noise levels used in this study. The same number of acquisitions acquired on an existing CT system using the lowest tube current at about 10 mA and a rotation time of 1 s will result in an averaged image representing 1280 mAs, which should well be approximated as noise-free. The size of the x-ray focal spot would have changed between acquisitions using 10 and 1280 mA, where the latter tube output is about max for such a CT system. A change from a small to a large focal spot will cause degradation in the spatial resolution and will be analyzed as a nonlinear distortion in an ${\mathrm{NLD}}_{\text{object}}^{\prime }$ image. Hence, an analysis of the nonlinear distortion using ${\mathrm{NLD}}_{\text{object}}^{\prime }$ image may best be performed with the same size of the focal spot. The available tube current range for a small size of the x-ray focal spot can be about between 10 and 400 mA. Thus if the ${\mathrm{NLD}}_{\text{object}}^{\prime }$ image was acquired using these limits of the tube current and repeated to achieve an averaged image representing 1600 mAs using a rotation time of 1 s, the theoretic acquisition time of all images required for the calculation would have been <3  min (160  s+4  s=2  min 44  s) plus perhaps some tube cooling time.

It may not be possible to estimate an approximation for the ${\mathrm{NLD}}_{\text{noise}}$ series as noise is randomly generated and cannot be easily approximated without the object being present. In the estimation of ${\mathrm{NLD}}_{\text{noise}}$, the noise is isolated before and after reconstruction, i.e., reconstructed without and with the object being present. Further, the noise sinograms are estimated by subtracting the average of the noisy sinograms. Hence, the estimation of the object will not be perfect but also consists of the noise variations originating from the sampled acquisitions. In practice, the test object will further be affected by a range of other types of variations, such as electronic noise, scatter, vibrations of the CT, and a polychromatic x-ray spectrum. However, as the systematic part (the estimation of the test object) is subtracted, only the random fluctuations (the estimation of the noise) are left, both before and after the reconstruction. Hence, the ${\mathrm{NLD}}_{\text{noise}}$ will indicate the difference in how the reconstruction algorithm handles these fluctuations when an object is present or not. For a linear CT system, the variations will be reconstructed equally and ${\mathrm{NLD}}_{\text{noise}}$ will be close to zero, which was indicated by the FBP algorithm in this study.

It may not be obvious how the content of the ${\mathrm{NLD}}_{\text{object}}$ or ${\mathrm{NLD}}_{\text{noise}}$ images should be interpreted. However, noise-dependent smoothing of the reconstructed object can be indicated by the structures of distortion in the ${\mathrm{NLD}}_{\text{object}}$ or ${\mathrm{NLD}}_{\text{noise}}$ image. Further, dark areas corresponding to the reconstructed object can indicate degradation of the contrast of these objects. Improved spatial resolution or contrast enhancement cannot be indicated as this type of image shows the difference when reconstructing an object in low versus high noise. Hence, it is unlikely that a noise reduction algorithm will increase the image quality as the noise is increased. Thus bright or dark regions in the ${\mathrm{NLD}}_{\text{object}}$ or ${\mathrm{NLD}}_{\text{noise}}$ images that do not correspond to the reconstructed object could indicate that an additional object or texture has been added to the image by the nonlinear noise reduction algorithm due to the noise or the reconstructed object. Accordingly, the ${\mathrm{NLD}}_{\text{object}}$ and ${\mathrm{NLD}}_{\text{noise}}$ images visualize undesirable effects of the algorithm. An ideal noise reduction algorithm would have generated distortion images without structures or unwanted alterations in the noise texture. However, it could be possible to approximate the CT system as linear if the ${\mathrm{NLD}}_{\text{object}}$ and ${\mathrm{NLD}}_{\text{noise}}$ only indicated stochastic variations. Further, such approximation may only be valid for the object used in the analysis. Although four CNRs were considered in this study, the ${\mathrm{NLD}}_{\text{object}}$ and ${\mathrm{NLD}}_{\text{noise}}$ images can be used to visualize the nonlinear properties of a noise reduction algorithm at any noise level, to indicate the noise dependence of the algorithm. A deep learning algorithm can train a neural network to reduce noise in high-noise images by comparing them to a low-noise image of the same object. The NLD image would probably show less distortion for the noise levels at which the algorithm was trained, and the distortion may be increased for the noise levels far from the trained levels. However, other types of noise reduction algorithms, such as model-based iterative reconstruction techniques, would have probably induced distortions similar to the algorithms tested in this study.

The distribution image of the nonlinear distortion was contaminated by noise as each estimation of the distortion contained both distortion and noise. The systematic nonlinear distortion described by the ${\mathrm{NLD}}_{\text{object}}$ was not varied a lot across space between the 5th- and 95th-percentile images. Hence, the difference between these images reflected the noise distribution more than the nonlinear distortion as the Hamming filter for the FBP algorithm did not reduce noise to the same extent as the nonlinear noise reduction algorithms. However, the distortion variation across space could be related to the noise distribution and could indicate if the amplitude of the nonlinear distortion would have been larger than the noise. Further, the more detailed analysis of the costa edge against the lung indicated that the systematic nonlinear distortion was comparable to the noise level. However, the nonlinear distortion and noise in a single image could be higher or lower at specific positions in space depending on the object and noise variations. Hence, the risk of a nonlinear noise reduction algorithm obscuring the pathology due to distortion may still be difficult to assess. However, the NLD methodology might help with understanding many properties of various nonlinear algorithms in the future.

The method presented here, using ${\mathrm{NLD}}_{\text{object}}$ and ${\mathrm{NLD}}_{\text{noise}}$ images, was inspired by the methodology of DPS, which relates the distorted signal to the original signal, i.e., the object before reconstruction.^15^^,^^16^ Such an analysis will handle all of the distortion effects of a CT system, including aliasing and other nonlinear distortions due to geometrical inconsistency. However, when analyzing nonlinear noise reduction algorithms, it may be better to exclude distortion effects that do not originate from the noise reduction algorithm. This was achieved in this study by relating the nonlinear distortion effect to that between two different noise levels after reconstruction. Hence, both reconstructed images have been affected by the same geometrical distortion. The DPS can also be estimated without geometrical distortion using a method similar to the present method, but that has yet to be tested. Further, this study showed how to separate the distortion of the signal from the distortion of the noise. Hence, the general theory of the DPS estimation applied in the image domain and on a typical CT image with anatomical structures will result in an image containing the sum of the ${\mathrm{NLD}}_{\text{object}}$ and an average of the ${\mathrm{NLD}}_{\text{noise}}$ series.

Solomon et al.^27^ proposed a method that can assess noise properties using anthropomorphic phantoms and compared nonlinear noise reduced images with images reconstructed using FBP. Their method clearly visualized the noise reduction of a nonlinear algorithm to be dependent on the object by isolating the noise from the object after the reconstruction. Further, the subtraction of the noise magnitude of one image from another reconstructed with a different algorithm may show the location of the noise difference between the algorithms. However, the effects of nonlinear distortion may be altered or completely concealed by the difference in resolution or noise properties of the reconstruction algorithms being compared. Hence, even if the method is similar to the present method, the focus of the studies was not the same because, in such a method, the nonlinear effects are not isolated even if the compared algorithm is FBP, i.e., is linear. In contrast, the method described in this study isolates the nonlinear distortion using the same nonlinear algorithm. Thus the analysis is not influenced by or dependent on the performance of a second algorithm. Hence, the properties of the nonlinear algorithm may be analyzed independently. Further, the focus of the ${\mathrm{NLD}}_{\text{object}}$ and ${\mathrm{NLD}}_{\text{noise}}$ images is not to estimate noise reduction but the distortion effect caused by the nonlinear noise reduction algorithm.

The observed degradation of the resolution and contrast in nonlinear reconstructions as the noise increases is supported by previous findings using lesion simulation in patient images and with findings in studies using the task transfer function (TTF).^10^^,^^28^ The lesion simulation study described how a nonlinear noise reduction algorithm of an existing CT system affected the reconstruction of a single simulated lesion at different noise levels.^28^ The present method was developed to trace the same nonlinear effect but for the whole patient content. Further, the method applied on an existing CT system would not need to simulate objects or noise. In the case of the TTF method, it has recently been modified to be analyzed in clinical images and, in combination with the noise power spectrum, has been used to estimate the detectability index ($d^{\prime }$) of specific tasks.^13^^,^^14^ The $d^{\prime }$ is useful for predicting the correlation between the image quality and dose reduction in optimization of CT examinations. However, the appearance of the ${\mathrm{NLD}}_{\text{object}}$ and ${\mathrm{NLD}}_{\text{noise}}$ images may be a complement to the $d^{\prime }$ estimation by reflecting the overall nonlinear effect visually. Thus it would indicate whether there is a risk that the diagnostic task has been affected by the noise reduction algorithm and needs further investigation. The numerical figure of merit could be developed from the ${\mathrm{NLD}}_{\text{object}}$ and ${\mathrm{NLD}}_{\text{noise}}$ images as a measure of the effect in diagnostic tasks. For example, such a figure of merit could analyze the correlation of the distortion to the object and may further be used to indicate if the nonlinear noise reduction algorithm may be generally handled as a linear algorithm.

There are obvious limitations with this study. In addition to the lack of a specific figure of merit in this study, the simulation of an ideal CT system was another limitation as noise reduction algorithms on existing CT systems were not analyzed with the proposed method. Further, the investigated nonlinear noise reduction algorithms were only applied on the sinogram data and were not integrated in the updating procedure of the pixel values. However, the focus of this study was not to develop a nonlinear noise reduction algorithm but a method to visualize the effect of it. Furthermore, assessment on a patient should theoretically be possible although not convenient or suitable with conventional CT systems. However, the first photon-counting CT systems have been installed at radiological departments around the world. The generation of ${\mathrm{NLD}}_{\text{object}}$ and ${\mathrm{NLD}}_{\text{noise}}$ images without the need for repeating the acquisition should be possible on these systems if equipped with a feature to time integrate the acquisition at various intervals (similar to modern single photon energy CT systems) as a new noise will randomly be obtained at each time interval. Subtraction in the projection domain would still be needed to generate ${\mathrm{NLD}}_{\text{noise}}$ images. Nevertheless, the use of anthropomorphic phantoms may be sufficient to gain a better understanding of how the anatomy of a patient and/or pathology is affected by nonlinear noise reduction algorithms in conventional CT systems. Furthermore, the present method may be applied to any imaging modality involving nonlinear denoising algorithms, such as compressed sensing in magnetic resonance imaging or deep learning reconstruction in positron emission tomography.

## 5 Conclusions

We have described a method of analyzing nonlinear noise reduction algorithms in CT imaging independently of other algorithms by estimating and visualizing the ${\mathrm{NLD}}_{\text{object}}$ and ${\mathrm{NLD}}_{\text{noise}}$. The ${\mathrm{NLD}}_{\text{object}}$ image describes how objects are distorted due to noise by visualizing the degradation of resolution and contrast of an arbitrary object, whereas the ${\mathrm{NLD}}_{\text{noise}}$ series indicates how the noise is affected by the noise reduction of a nonlinear reconstruction algorithm in the presence of an object. The induced distortion may be both stochastic and correlated to the object, and analyzing the latter may be more critical. The absence of nonlinear distortion may be used as a measure of robustness of the denoising algorithm. Further analysis using the proposed method may improve our understanding of the effects of noise reduction algorithms on image quality and their dependence on anatomical structures and noise.
